# Evaluation of Sample Preparation Methods for Inter-Laboratory Metabolomics Investigation of *Streptomyces lividans* TK24

**DOI:** 10.3390/metabo10090379

**Published:** 2020-09-22

**Authors:** Howbeer Muhamadali, Kenneth Simoens, Yun Xu, Bart Nicolai, Kristel Bernaerts, Royston Goodacre

**Affiliations:** 1 Department of Biochemistry and Systems Biology, Institute of Systems Molecular and Integrative Biology, University of Liverpool, Biosciences Building, Liverpool L69 7ZB, UK; Howbeer.Muhamad-Ali@liverpool.ac.uk (H.M.); yun.xu@liverpool.ac.uk (Y.X.); 2 School of Chemistry, Manchester Institute of Biotechnology, University of Manchester, Manchester M1 7DN, UK; 3 Bio- and Chemical Systems Technology, Reactor Engineering and Safety Section, Department of Chemical Engineering, KU Leuven (University of Leuven), Leuven Chem&Tech, Celestijnenlaan 200F Box 2424, 3001 Leuven, Belgium; kenneth.simoens@kuleuven.be (K.S.); kristel.bernaerts@kuleuven.be (K.B.); 4 Division of Mechatronics, Biostatistics and Sensors (MeBioS), Department of Biosystems (BIOSYST), KU Leuven (University of Leuven), Willem de Croylaan 42 Box 2428, 3001 Leuven, Belgium; bart.nicolai@kuleuven.be

**Keywords:** metabolomics, *Streptomyces*, biotechnology, GC-MS, sample preparation

## Abstract

In the past two decades, metabolomics has proved to be a valuable tool with many potential applications in different areas of science. However, there are still some challenges that need to be addressed, particularly for multicenter studies. These challenges are mainly attributed to various sources of fluctuation and unwanted variations that can be introduced at pre-analytical, analytical, and/or post-analytical steps of any metabolomics experiment. Thus, this study aimed at using *Streptomyces lividans* TK24 as the model organism in a cross-laboratory experiment in Manchester and Leuven to evaluate the reproducibility of a standard sample preparation method, and determine the optimal sample format (cell extract or quenched biomass) required to preserve the metabolic profile of the cells during cross-lab sample transportation and storage. Principal component analysis (PCA) scores plot of the gas chromatography-mass spectrometry (GC-MS) data from both laboratories displayed clear growth-dependent clustering patterns which was in agreement with the Procrustes analysis findings. In addition, the data generated in Manchester displayed tight clustering of cell pellets (quenched biomass) and metabolite extracts, confirming the stability of both sample formats during the transportation and storage period.

## 1. Introduction

*Streptomyces* spp. are unique amongst the bacteria as they form filamentous mycelia and secrete a large number of natural products (NPs) including hydrolytic enzymes and secondary metabolites with key pharmaceutical and industrial applications [1], such as antibacterial molecules [2], anticancer agents [3], immunosuppressants [4], and insecticides [5]. It is such ability of these bacteria that has also made them an ideal host for the production of various recombinant proteins. However, one of the main obstacles for using these organisms is that most of their NPs are produced in very low titers, or their biosynthetic gene clusters are cryptic under laboratory conditions. Recent studies have demonstrated the importance of multi-omics approaches for better understanding and establishing the connection between gene expression and metabolism [6,7], which may provide new opportunities for the discovery of novel NPs [8,9]. In addition, the application of a top-down approach, starting with metabolomics assessment of a bioprocess and subsequently moving to genomics methods [10], utilizing a priori knowledge of the whole metabolic network and regulatory systems, may allow for the detection of target pathways and rate-limiting metabolites at key fermentation times, and support future metabolic-engineering strategies which are needed within a synthetic biology framework [11].

Since the term metabolome was first proposed by Oliver et al. in 1998 [12], the application of mass spectrometry-based metabolomics approaches in the field of microbiology has received increasing interest, which is also evident from the exponential rise in the number of publications in this area. Metabolomics usually covers areas of science focusing on the detection and quantification of low molecular weight molecules (metabolites) in a biological system via different approaches, such as targeted analysis, untargeted analysis, fingerprinting, and footprinting [13,14]. These approaches have been applied in various areas of microbiology including within the industrial [15,16], environmental [17,18], and medical fields [19,20]. Nevertheless, untargeted metabolomics has become the most popular approach of them all [21], as it does not require a priori hypothesis, it allows for the detection of as many metabolites as possible, and it also provides a snapshot of the metabolic state of a biological system. However, one of the main challenges of untargeted metabolomics, especially in multicenter and large clinical studies, is preserving this metabolic snapshot and preventing and/or minimizing unwanted variations and artifacts that may lead to false discoveries and thus incorrect hypothesis generation [22].

Generally untargeted metabolomics studies involve three main steps including: (1) pre-analytical (experimental design, sample collection, sample treatment, and storage), (2) analytical (sample analysis and data collection), and (3) post-analytical (deconvolution, data processing, and biological interpretation). In most metabolomics studies the pre-analytical step is commonly regarded as the easiest step; however, as the metabolome is highly dynamic and prone to change, the pre-analytical step is in fact the most crucial step as it defines the sample quality and reproducibility, and therefore it needs to be correctly designed and tightly regulated [19,23,24]. Examples of such unwanted variations, potentially introduced at the pre-analytical step, include differences in reagent batches, container material, sample handling and processing protocols, and storage conditions [21].

Koning and colleagues reported the turnover rate of cytosolic glucose in *Saccharomyces cerevisiae* to be around 1 mM s^−1^ [25], which highlights the importance of efficient sample quenching and fixation protocols required during the pre-analytical step [26]. It is perhaps also worth noting that even after the quenching step, unless the metabolites are extracted and analyzed immediately, the stability of the samples can still be affected due to various factors such as enzymatic degradation and long-term storage as well as the storage temperature [21]. In addition, the chemically-diverse nature of metabolites means that many are labile and so may be non-enzymatically transformed during storage [27].

**Figure 1:**
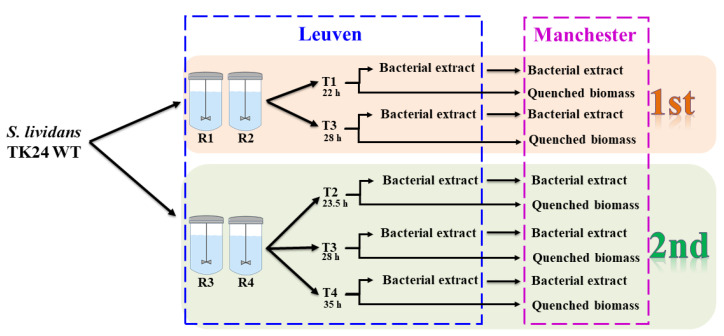
Overview of the experimental design. R = reactor and T = time of sampling.

This study aimed at assessing the sample preparation protocols required for untargeted metabolic profiling of *S. lividans* TK24 [28], in an inter-lab study between Manchester and Leuven, considering various biological and analytical variabilities. All cells were grown in the Leuven laboratories as duplicates in two separate bioreactors, and the experiment was repeated twice (Figure 1). At various timepoints, three sets of samples were prepared, including two sets of bacterial extracts and one set of quenched intact cells (cell pellet). The first set of the bacterial extracts were analyzed in the Leuven laboratories using their GC-MS setup (outlined below), while the remaining sets of bacterial extracts along with the quenched cell pellets were shipped on dry ice to Manchester. This allowed for comparison and assessment of the stability and reproducibility of the samples (metabolic profiles) during the cross-lab shipping process. In addition, to investigate further the stability of the metabolites and verify the optimal sample format required for long term storage after shipping, all samples arriving in the Manchester laboratories were stored immediately at −80 °C for six months before being processed and analyzed.

## 2. Results

### 2.1. GC-MS Metabolic Profiles

In order to investigate the effects of transport and storage of bacterial samples for metabolomics, we designed a study where a set of identical samples of *S. lividans* were generated in Leuven. Subsequently: (i) the bacterial extracts generated in Leuven were analyzed immediately in Leuven and (ii) the same bacterial extracts, as well as quenched biomass, were shipped and stored at −80 °C for 6 months in Manchester, before being analyzed in Manchester. In Manchester, the same bacterial extracts as investigated in Leuven were analyzed, as well as metabolite extracts from the cell pellets, using the same 100% cold (−45 °C) methanol solution for small molecule extraction as performed in Leuven.

It is common in metabolomics that different laboratories undertake contrasting analytics. Our two laboratories have individually optimized our GC-MS methods and thus we thought this was important to include in this study. The salient differences are highlighted below:

- Derivatization: both labs used the same methoximation step, but the silylation reagent was MSTFA (Manchester) or BSTFA (Leuven).
- *GC-MS*: Agilent 6890N GC coupled to a Leco Pegasus III ToF-MS (Manchester) versus an Agilent 7890A GC with an Agilent 5975C VL MSD system (Leuven).
- Internal standard: this was either succinic-*d*_4_ acid (Manchester) or phenyl β-d-glucopyranoside (Leuven).
- Metabolite identification: Level 1 of metabolomics standards initiative (MSI) guidelines to an in-house library (Manchester) versus matching to the Golm library for MSI Level 2 (Leuven).

**Figure 2:**
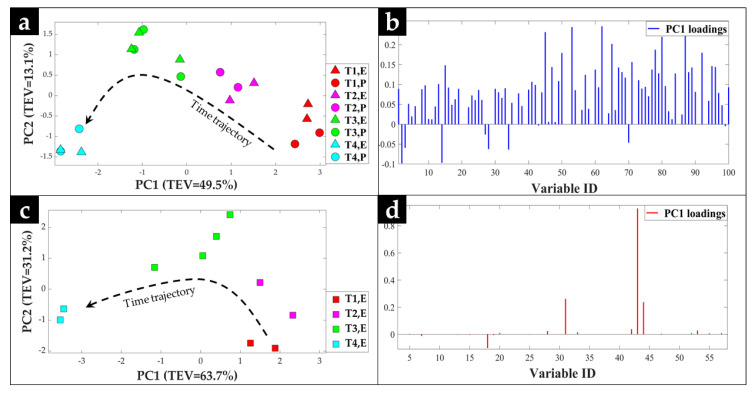
PCA scores and loadings plots of the GC-MS data acquired from samples analyzed in (**a**,**b**) the Manchester, and (**c**,**d**) the Leuven laboratories, respectively. Different colors indicate the various timepoints, while the symbols represent the sample sets, Manchester extracts (triangle, E), Manchester cell pellet (circle, P), and Leuven extract (square, E). The arrows illustrate the main trajectories with respect to time. The values in parentheses are total explained variance (TEV).

The principal component analysis (PCA) scores plot of the pre-processed GC-MS data generated in Manchester (Figure 2a) displayed clear separation of the samples based on their corresponding incubation time, according to the PC1 axis with a total explained variance (TEV) of 49.5%, which is not surprising considering the metabolic changes of *S. lividans* throughout its growth in liquid cultures. In addition, the data points corresponding to the cell extract and pellet samples, from individual biological replicates, were tightly clustered, which also suggested that the metabolic profiles of the cells in both sample formats were comparable, and more importantly conserved during the transportation and storage periods in this study.

**Figure 3:**
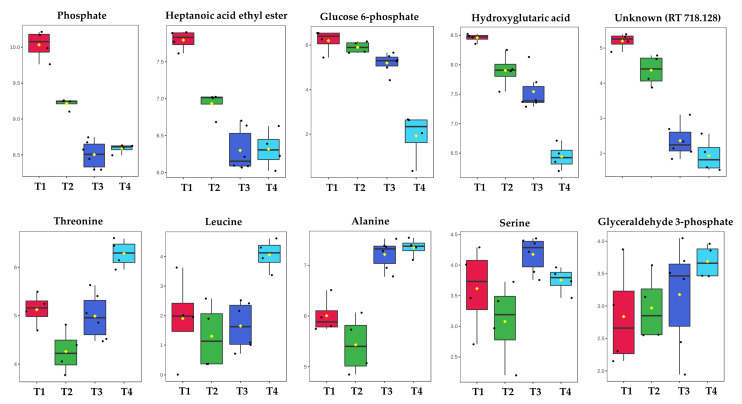
Relative peak areas of the significant metabolites identified by PCA of the GC-MS data acquired from the samples analyzed within the Manchester laboratories. Different colors indicate the various timepoints, T1 (22 h, red), T2 (23.5 h, green), T3 (28 h, blue), and T4 (35 h, cyan).

**Figure 4:**
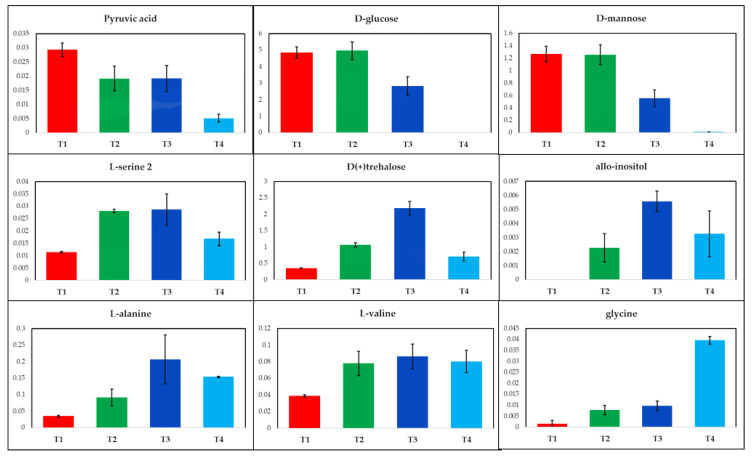
Relative peak areas of the significant metabolites identified by PCA of the GC-MS data acquired from the cell extracts analyzed within the Leuven laboratories. Different colors indicate the various timepoints, T1 (22 h, red), T2 (23.5 h, green), T3 (28 h, blue), and T4 (35 h, cyan).

PCA scores plot of the GC-MS data generated in Leuven (Figure 2c), displayed similar trends, exhibiting a gradual time-dependent change in the metabolic profiles of the cells, where samples clustered mainly according to their corresponding incubation times across the PC1 axis with a TEV of 63.7%; this greater TEV was likely to be a function of the number of metabolite features used for PCA which for Leuven was half that (*n* = 31) compared to Manchester (*n* = 85). PC1 loadings plots of both Manchester (Figure 2b) and Leuven (Figure 2d) GC-MS data were employed to identify the most significant metabolites contributing to the observed clustering patterns. Relative peak areas of the top nine significant metabolites were identified and plotted as box and whisker plots and bar charts for all samples analyzed in both Manchester (Figure 3) and Leuven (Figure 4), respectively. The full list of the top significant metabolites identified in both laboratories is provided in Tables S1 and S2 in the Supplementary Materials.

The levels of glucose 6-phosphate, heptanoic acid ethyl ester, hydroxyglutaric acid, and phosphate for samples analyzed in Manchester exhibited a gradual decrease with time, while some of the amino acids including alanine, threonine, and leucine, displayed the opposite trend and showed increasing levels with time. 

The samples analyzed in Leuven also displayed a decreasing trend for some of the main metabolites found in the glycolysis pathway, including glucose and pyruvic acid, with increasing incubation time (Figure 4). The cell extracts analyzed in Leuven also highlighted the accumulation of some amino acids with increasing incubation time, including serine, valine, glycine, and alanine (Figure 4). Trehalose and allo-inositol were also amongst the metabolites that displayed an increasing trend.

**Figure 5:**
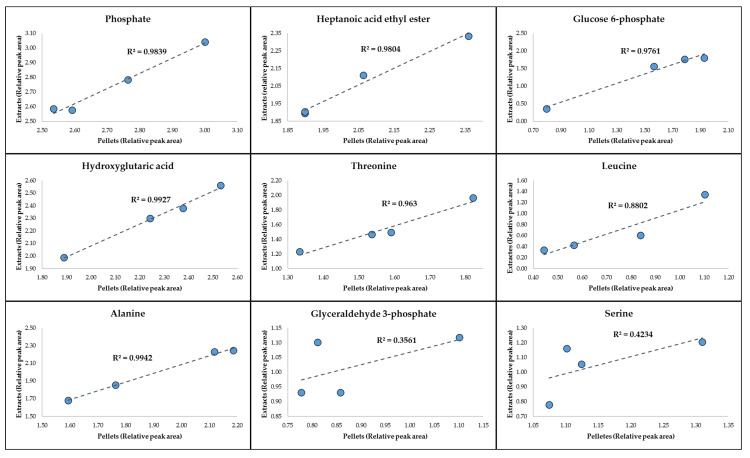
Plots of GC-MS relative peak areas of the significant metabolites detected in the cell pellets vs. those detected in cell extracts.

To further evaluate the stability of the detected metabolites during the cross-lab transportation and storage period (6 months at −80 °C), the level of the significant metabolites in all samples analyzed in Manchester was compared by plotting the detected peak area of the metabolites in the cell extract against those of the pellet samples (Figure 5).

Overall, the results (Figure 5) displayed a clear correlation between the levels of seven of the metabolites detected in the cell extracts compared to those of the cell pellets, with *R*^2^ ranging from 0.88 to 0.99. By contrast, the relative peak areas of glyceraldehyde 3-phosphate and serine were not as comparable possibly due to the lower level of these metabolites detected in these samples. Notwithstanding, the overall detected changes of these two metabolites over time were the same and displayed similar trends in both the cell extracts and cell pellet samples, individually.

### 2.2. Procrustes Analysis of the GC-MS Data 

**Figure 6:**
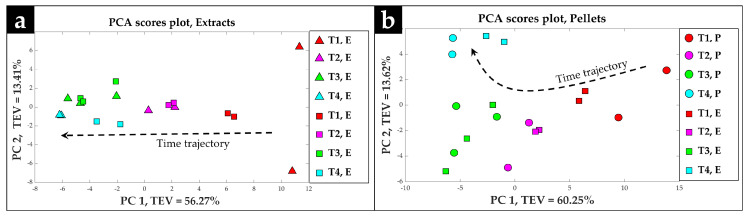
Procrustes analysis performed on the PCA scores of the GC-MS data generated from (**a**) cell extracts in both Manchester and Leuven and (**b**) cell pellets in Manchester and cell extracts in Leuven. Different colors indicate the various timepoints, while the symbols represent the sample sets, Manchester extracts (triangle, E), Manchester cell pellet (circle, P), and Leuven extract (square, E). The arrows illustrate the main trajectories with respect to time. The values in parentheses are total explained variance (TEV).

The PCA score plots of the data from Leuven and Manchester looked very similar and we sought to confirm this more objectively. Procrustes analysis in simple terms can be defined as a multivariate exploratory technique that can be used for comparison and matching of data matrices [29]. The algorithm usually takes one configuration as a target and tries to fit the other to it by applying three admissible transformations (translation, rotations, and re-scaling). To compare and assess the GC-MS data of both cell extract and pellet samples generated in Manchester with the cell extracts in Leuven, the PCA scores of both datasets were subjected to Procrustes analysis to evaluate the observed clustering patterns (Figure 6).

For cell extract samples, Procrustes analysis were applied to the first three PCs of PCA scores of the Manchester data set which accounted for 87.6% of the total explained variance (TEV) and first four PCs of the Leuven data set which accounted for 86.3% of the TEV. The resulting Procrustes distance was 0.3842. Comparing this distance to the corresponding NULL distribution obtained from 1000 randomized permutation tests, an empirical *p*-value of 0.002 was obtained (i.e., out of 1000 permutations, only two cases had obtained a Procrustes distance lower than 0.3842). This strongly suggests that the observed similarity was highly significant. The Procrustes-rotated first two PCs of PCA scores of the Leuven data set was superimposed on top of those of the Manchester data set and is shown in Figure 6a. The results displayed a clear time-dependent clustering of the data points according to the PC1 axis with a TEV of 56.27%. The cell extracts for each timepoint, analyzed in Manchester (triangle symbols) clustered accordingly with its counterpart in Leuven (square symbols). However, it should be noted that the cell extracts analyzed in Manchester at the first timepoint displayed a large variation and were separated according to the PC2 axis with a TEV of 13.41%.

For the *S. lividans* pellet samples, Procrustes analysis was applied to the first three PCs of both Leuven and Manchester data sets which accounted for 85.8% and 84.2% of TEV, respectively. The calculated Procrustes distance was 0.3932 and the empirical *p*-value < 0.001 was obtained when this distance was compared to the corresponding NULL distribution (i.e., out of 1000 permutations, not a single case had obtained a lower Procrustes distance). The Procrustes-rotated PCA scores plot in which the Procrustes-rotated first two PCs of Leuven data (square symbols) was superimposed on top of the first two PCs of Manchester data (circle symbols) is shown in Figure 6b. A clear separation and clustering of the datapoints based on the corresponding incubation times, according to the PC1 axis with a TEV of 60.25% was also observed.

## 3. Discussion

Due to the significant impact of microorganisms on our everyday life and the wide range of applications of their NPs and metabolites, microbial metabolomics has attracted a lot of interest with promising results. However, to acquire meaningful metabolomics data, appropriate sample-preparation protocols are required to maintain the stability of the metabolome. Thus, this study aimed at assessing two common sample formats, quenched cell pellets and extracted metabolites, for their reproducibility and efficiency in preserving the metabolic profiles of the cells during cross-laboratory transportation and long-term storage at −80 °C.

The most significant metabolites highlighted by the PCA loadings plots in all three sets of samples (Figure 2b,d) were mainly sugars and amino acids, which is in agreement with our previous findings [28,30]. Amongst these were glucose, glucose 6-phosphate, and pyruvic acid that displayed decreasing trends with time (Figure 3 and Figure 4). This is perhaps not surprising considering that *S. lividans* can uptake the glucose provided in the medium as the sole carbon source [28,31], which can then be passed down through glycolysis and is initially converted to glucose 6-phosphate and at the end of glycolysis to pyruvate (the glycolytic intermediates are rarely detected as they are at very low concentrations). With increasing incubation time and biomass, glucose availability in the medium will be limited, which will in turn contribute to lower levels of the aforementioned metabolites at later timepoints. Alanine was amongst the amino acids that displayed increasing trends with time in all sample sets. Such changes were also detected in our previous study [28], which could be due to a carbon overflow and a bottleneck between glycolysis and the TCA cycle resulting in the redirection of the carbon flow to alanine through conversion of pyruvate by the alanine dehydrogenase enzyme. Another metabolite that was identified as significant in Leuven-cell extracts was trehalose (Figure 4), which also displayed an increasing trend with time. In some bacterial cells, trehalose accumulation is linked to osmotic stress; however, accumulation of trehalose seems to be widely spread among *Streptomyces* species, which suggests the significant role of this metabolite in these bacteria [32]. Whilst some studies link trehalose to germination and carbohydrate storage in *Streptomyces*, others suggest the contribution of this metabolite to protection and structural integrity of the cell membrane under stress conditions such as heat and desiccation [33,34]. However, microscopic investigation of the samples at later timepoints displayed the presence of spores, which could be a contributing factor to the higher levels of trehalose detected in these samples.

Overall, the PCA score plots of the GC-MS data collected from all three sample sets (Figure 2a,c), Manchester pellets, extracts, and Leuven extracts, were in very good agreement and displayed comparable clustering patterns according to the growth profile of *S. lividans* at different time points. These findings are highly encouraging considering: (1) the complex life cycle of *Streptomyces* [35]; (2) the cells used in this study were grown across four separate bioreactors using different starting inocula; (3) the pre-analytical sample preparation steps was carried out in different laboratories; (4) the samples were analyzed on two separate instruments from different manufacturers with varying sensitivity and detection limits; and (5) these samples were analyzed 6 months apart.

More importantly, comparison of the GC-MS relative peak areas of the top significant metabolites detected in the cell pellets with those of the cell extracts (Figure 5), also confirmed the overall reproducibility and stability of the metabolic profiles of the samples during the transportation and storage period in both sample formats (cell extracts and pellets). Although most of the significant metabolites displayed a linear trend, the detected levels of serine and glyceraldehyde 3-phosphate was fluctuating between the cell extracts and cell pellets (Figure 5), which could be due to overlapping peaks or lower signal intensity of these peaks and consequently higher interference from background noise.

In conclusion, the results presented in this study indicate that standardization of protocols is critical, and if appropriate sample-preparation protocols are designed, tightly regulated, and followed, both dried cell extracts and quenched cell pellets can be used to acquire consistent and comparable metabolic profiles.

## 4. Materials and Methods

All chemicals were of analytical grade and purchased from Sigma Aldrich, unless stated otherwise.

### 4.1. Bacterial Strain and Growth Condition

The strain used in this study was *Streptomyces lividans* TK24 [28].

All cells were grown in minimal medium (10 g/L glucose, 3 g/L (NH_4_)_2_SO_4_, 2.6 g/L K_2_HPO_4_, 1.8 g/L NaH_2_PO_4_, 0.6 g/L MgSO_4_.7H_2_O, 1 mg/L ZnSO_4_.7H_2_O, 1 mg/L FeSO_4_.7H_2_O, 1 mg/L CaCl_2_, 1 mg/L MnCl_2_.4H_2_O) supplemented with 500 µL/L Y-30 antifoam, using a parallel bioreactor system (DASGIP, Eppendorf, Jülich, Germany) with a working volume of 1 L and controlling: pH at 6.8, temperature at 30 °C, stirring at 500 rpm, and aeration at 60 sL/h.

Biomass for inoculation of the bioreactors was obtained from a two-step preculture starting with dispensing a loop of frozen mycelium from −80 °C storage in 100 mL of phage medium (10 g/L glucose, 5 g/L tryptone, 5 g/L yeast extract, 5 g/L Lab Lemco Powder, 0.74 g/L CaCl_2_.2H_2_O, 0.5 g/L MgSO_4_.7H_2_O with pH 7.2) in a 250 mL Kleinfeld baffled shake flask and incubated at 30 °C for 72 h under moderate stirring. The resulting preculture is harvested by centrifugation (3200× *g* for 10 min at 30 °C) and diluted with ratio 1 to 4 in fresh phage medium followed by incubation for 24 h under the same conditions as stated above. A total of 25 mL preculture per liter reactor medium was used to seed the bioreactor preceded by concentrating and washing the biomass by means of repeated centrifugation and resuspending of the bacterial pellet in fresh minimal media.

Dry cell weight (DCW) measurements were obtained by centrifugation of 10 mL culture, resuspending the pellet in water and filtering under vacuum over a pre-dried and pre-weighted filter (Porafill Cellulose Mixed Esters, 0.2 µm pore size, Macherey-Nagel GmbH & Co. KG, Dueren, Germany). The filter with biomass was dried overnight at 105 °C and weighed again.

### 4.2. Quenching and Extraction

Samples (4 mL) taken at different timepoints (Figure 1) were quenched immediately by adding 8 mL of cold (−45 °C) 60% methanol/water solution followed by centrifugation at 3200× *g* for 10 min at −9 °C, as described previously [28,36]. After removing the supernatant, all cell pellets were snap frozen in liquid nitrogen for 1 min and stored at −80 °C until further analysis. In both laboratories, Manchester and Leuven, internal metabolites were extracted by freeze−thaw method following a standard step-by-step protocol [36], using 100% cold (−45 °C) methanol solution. Aliquots (50 µL) from all cell extracts were combined into one tube to be used for quality control (QC) purposes. Internal standard solution (0.2 mg/mL of glycine-*d_5_* and succinic-*d_4_* acid) was added (100 µL) to all cell extracts analyzed in Manchester, before being dried at room temperature overnight using a speed vacuum concentrator (Concentrator 5301, Eppendorf, Cambridge, UK).

For all samples analyzed in Leuven, an internal solution (291 mg/mL phenyl-beta-glucopyranoside) was added (45 µL) to the cell extract, which subsequently was dried in a speed vacuum concentrator (Centrivap, Labconco, Kansas City, MO, USA) for 8 h at 20 °C and preserved at −4 °C under vacuum before being stored at −80 °C.

### 4.3. Derivatization

All samples analyzed in Manchester were derivatized following a two-step process [37,38]: (1) oximation, using methoxiamine hydrochloride in pyridine, followed by (2) silylation, using *N*-Methyl-*N*-(trimethylsilyl) trifluoroacetamide (MSTFA). For samples analyzed in Leuven, *N*,*O*-Bis(trimethylsilyl)trifluoroacetamide (BSTFA) was used in the second step of the derivatization process.

### 4.4. GC-MS Analysis

In Manchester, all samples were analyzed on a Leco Pegasus III mass spectrometer (St. Joseph, MO, USA) coupled to an Agilent 6890N GC oven with a split/splitless injector and Agilent LPD split-mode inlet linear (Agilent Technologies, Stockport, UK), using a Gerstel MPS-2 autosampler (Gerstel, Baltimore, MD, USA). Injection parameters followed that of our previously-published data [39]. Sample analysis in Leuven was carried out on an Agilent 7890A GC coupled with an Agilent 5975C VL MSD system (Agilent Technologies, UK), using an Agilent HP-5MS column. A constant helium flow of 0.99 mL min^−1^ at a pressure of 7.5 psi, with a split ratio of 1:5. Temperature program: 2 min hold at 50 °C followed by 10 °C min^−1^ to 325 °C and 5 min hold.

### 4.5. Data Analysis

All GC-MS data were analyzed using MATLAB version 9 (The Mathworks Inc., Natwick, MA, USA). The collected GC-MS data of all the samples were normalized according to their corresponding DCW, and metabolite identification was carried out following the metabolomics standards initiative (MSI) guidelines [40]. Metabolite identification (Level 1 of MSI) for samples analyzed in Manchester was carried out using an in-house library [41], while the Golm library was used for putative metabolite identification (Level 2 of MSI) in Leuven [42]. Examples of the typical chromatograms detected in both laboratories are provided in Figure S1.

Peak areas of all detected metabolites were normalized by dividing them by those of their internal standards, the IS: 0.2 mg/mL succinic-*d_4_* acid for Manchester samples and phenyl-beta-glucopyranoside 291 µg/µL in methanol for Leuven samples, before being log_10_-scaled to ensure the metabolite peak areas were normally distributed.

All pre-processed data were subjected to exploratory analysis with principal component analysis (PCA) [43]. PCA is an unsupervised clustering method to reduce the dimensionality of the data without a priori knowledge of any grouping in the data, and to investigate the reproducibility of the sample formats and compare the clustering patterns between the two laboratories in this study.

Procrustes analysis [44] was applied to the PCA scores of the Leuven and Manchester data sets to measure the similarity of these two sets. Sufficient principal components (PCs) were retained so that the TEV of the two data sets was approximately the same. The similarity between the two data sets was calculated in terms of Procrustes distance which varied from 0 to 1. A distance of 0 means the two data sets were identical while 1 means there was nothing in common. A randomized permutation tests procedure called Procrustean test [45] was performed 1000 times to assess whether the probability of the calculated similarity was purely by chance or real.

The raw data supporting the conclusions of this article have been deposited to the EMBL-EBI MetaboLights database [46] (DOI: 10.1093/nar/gkz1019, PMID:31691833) with the identifier MTBLS2013. The complete dataset can be accessed here [47]. 

## Supplementary Materials

The following are available online at https://www.mdpi.com/2218-1989/10/9/379/s1, Table S1: List of the significant metabolites detected in Manchester laboratory, and identified using the PCA loadings plot. All identifications are based on minimum metabolite reporting standards, Table S2: List of the significant metabolites detected in Leuven laboratory, and identified using the PCA loadings plot. All identifications are based on minimum metabolite reporting standards, Figure S1: Typical GC chromatograms of *S. lividans* cell extracts detected via the GC-MS platforms employed at (a) Manchester and (b) Leuven laboratories.
