# Supplementary material for: Evaluation of Sample Preparation Methods for Inter-Laboratory Metabolomics Investigation of Streptomyces lividans TK24
Source: Metabolites. 2020 Sep 22;10(9):379. doi: 10.3390/metabo10090379 (PMC7569812; doi:10.3390/metabo10090379)
Supplement: Supplementary file 1 [file metabolites-10-00379-s001.pdf]

Evaluation of sample preparation methods for inter-laboratory metabolomics investigation of *Streptomyces lividans* TK24

Howbeer Muhamadali [1,2], Kenneth Simoens [3], Yun Xu [1,2], Bart Nicolai [4], Kristel Bernaerts [3] and Royston Goodacre [1,2,*]

[1] Department of Biochemistry and Systems Biology, Institute of Systems Molecular and Integrative Biology, University of Liverpool, Biosciences Building, Liverpool, L69 7ZB, UK; Howbeer.Muhamad-Ali@liverpool.ac.uk (H.M.); yun.xu@liverpool.ac.uk (Y.X.)

[2] School of Chemistry, Manchester Institute of Biotechnology, University of Manchester, Manchester, M1 7DN, UK

[3] Bio- and Chemical Systems Technology, Reactor Engineering and Safety section, Department of Chemical Engineering, KU Leuven (University of Leuven), Leuven Chem&Tech, Celestijnenlaan 200F box 2424, 3001 Leuven, Belgium; kenneth.simoens@kuleuven.be (K.S.); kristel.bernaerts@kuleuven.be (K.B.)

[4] Division of Mechatronics, Biostatistics and Sensors (MeBioS), Department of Biosystems (BIOSYST), KU Leuven (University of Leuven), Willem de Croylaan 42 box 2428, 3001 Leuven, Belgium; bart.nicolai@kuleuven.be (B.N.)

Correspondence: roy.goodacre@liverpool.ac.uk*

Abstract:

In the past two decades, metabolomics has proved to be a valuable tool with many potential applications in different areas of science. However, there are still some challenges that need to be addressed, particularly for multicenter studies. These challenges are mainly attributed to various sources of fluctuation and unwanted variations that can be introduced at pre-analytical, analytical, and/or post-analytical steps of any metabolomics experiment. Thus, this study aimed at using *Streptomyces lividans* TK24 as the model organism in a cross-laboratory experiment in Manchester and Leuven to evaluate the reproducibility of a standard sample preparation method, and determine the optimal sample format (cell extract or quenched biomass) required to preserve the metabolic profile of the cells during cross-lab sample transportation and storage. Principal component analysis (PCA) scores plot of the gas chromatography-mass spectrometry (GC-MS) data from both laboratories displayed clear growth-dependent clustering patterns which was in agreement with the Procrustes analysis findings. In addition, the data generated in Manchester displayed tight clustering of cell pellets (quenched biomass) and metabolite extracts, confirming the stability of both sample formats during the transportation and storage period.

Table S1. List of the significant metabolites detected in Manchester laboratory, and identified using the PCA loadings plot. All identifications are based on minimum metabolite reporting standards.

Variable ID	Metabolite ID	RI	RT	MSI Level	chEBI Code
3	Alanine_1096_2TMS	1095.1	362.128	1	16449
8	Heptanoic acid, ethyl ester_1208	1200.5	416.278	1	86618
14	Leucine_1306_2TMS	1286	460.128	1	25017
25	Phosphate_1372_4TMS	1350.2	493.078	1	18367
27	Serine_1397_3TMS	1369.6	503.078	1	17822
28	Threonine_1411_3TMS	1380.9	508.878	1	26986
41	2-hydroxyglutaric acid_1641_3TMS	1656.6	622.078	1	17084
65	Unknown	1953.4	718.128	4	-
70	Glyceraldehyde-3-phosphate_1834_3TMS	2039.9	742.628	2	17138
77	Glucose-6-phosphate_2368_6TMS	2244.7	800.678	2	14314

Table S2. List of the significant metabolites detected in Leuven laboratory, and identified using the PCA loadings plot. All identifications are based on minimum metabolite reporting standards.

Variable ID	Fiehn ID	RT	MSI Level	chEBI Code
5	Pyruvic acid	7.8867	2	32816
7	L-alanine	8.7065	2	16977
15	L-valine	10.415	2	16414
19	glycine	11.7094	2	15428
22	L-serine	12.4817	2	17115
43	D-glucose	18.9055	2	17634
44	D-mannose	19.0686	2	16024
49	allo-inositol	20.7143	2	22357
57	D(+)trehalose	26.148	2	27082

Code: ID, identifier on plots; RT, retention time; RI, retention index; MSI, Metabolomics Standards Initiative identification level; for chEBI codes see: https://www.ebi.ac.uk/chebi/

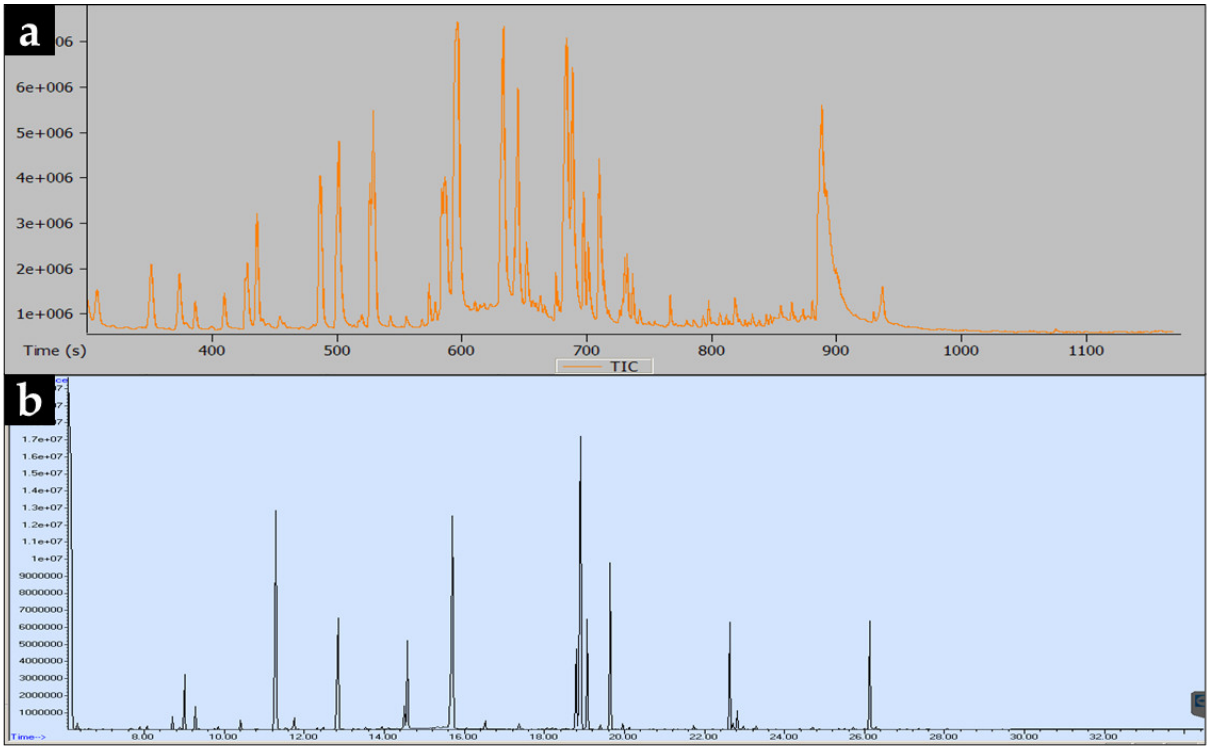

Figure S1. Typical GC chromatograms of *S. lividans* cell extracts detected via the GC-MS platforms employed at (a) Manchester and (b) Leuven laboratories.
